# Globally Guided Deep V-Network-Based Motion Planning Algorithm for Fixed-Wing Unmanned Aerial Vehicles

**DOI:** 10.3390/s24123984

**Published:** 2024-06-19

**Authors:** Hang Du, Ming You, Xinyi Zhao

**Affiliations:** 1 Sino-European Institute of Aviation Engineering, Civil Aviation University of China, Tianjin 300300, China; 176041203@cauc.edu.cn; 2 Shenyang Aircraft Design and Research Institute, Shenyang 110035, China; mywbdl@tju.edu.cn

**Keywords:** motion planning, deep V-Network, fixed-wing UAVs, globally path-guided

## Abstract

Fixed-wing UAVs have shown great potential in both military and civilian applications. However, achieving safe and collision-free flight in complex obstacle environments is still a challenging problem. This paper proposed a hierarchical two-layer fixed-wing UAV motion planning algorithm based on a global planner and a local reinforcement learning (RL) planner in the presence of static obstacles and other UAVs. Considering the kinematic constraints, a global planner is designed to provide reference guidance for ego-UAV with respect to static obstacles. On this basis, a local RL planner is designed to accomplish kino-dynamic feasible and collision-free motion planning that incorporates dynamic obstacles within the sensing range. Finally, in the simulation training phase, a multi-stage, multi-scenario training strategy is adopted, and the simulation experimental results show that the performance of the proposed algorithm is significantly better than that of the baseline method.

## 1. Introduction

With the development and wide application of UAV technology, fixed-wing UAVs have gradually become important tools in many fields due to their high maneuverability and survivability [1,2,3]. Unlike quadcopters, fixed-wing UAVs have huge advantages in endurance, flight speed, payload capacity, etc. [4,5], resulting in great potential in both civil and military applications [6]. Motion planning, as one of the key problems in flight control, is crucial for realizing safe, efficient, and autonomous flight missions. Collision avoidance is a key point in motion planning [7,8]. However, the flight characteristics of fixed-wing UAVs are very complex due to kinematic constraints [9]. Therefore, the goal of this paper is to learn a collision-free, safe, and fast motion planning method for fixed-wing UAVs that satisfies the kinematic constraints.

Unlike the path planning problem, kinodynamic motion planning for fixed-wing UAVs requires consideration of kinematic equations, kinematic constraints (e.g., obstacle avoidance), and kinodynamic constraints (e.g., velocity, acceleration, and turning radius constraints, etc.) in order to successfully reach the target location [10]. Although the traditional motion planning algorithm becomes less computationally practical for extremely dense scenarios, by simplifying the model, solvers enable real-time motion planning in many situations of interest.

A key challenge is to consider the second-order kinematics model and corresponding constraints of fixed-wing UAVs while achieving safe and fast motion planning in multi-obstacle (dynamic and static obstacles) environments. This is straightforward in a static environment, using first-order kinematics equations or simplifying the dynamics module, but the mixture of these factors makes it difficult to solve. Although existing studies have achieved some results in UAV path planning, it is still a worthwhile research problem [11,12].

In this work, we propose a hybrid A* deep V-network (HADVN) algorithm to realize collision-free motion planning for fixed-wing UAVs. In the proposed algorithm, a hierarchical two-layer motion planning framework is designed. First, considering the kinematic constraints of fixed-wing UAVs, such as the turning radius and the speed range, a global planner is designed based on to a modified hybrid A* search, providing a reference guidance with respect to static obstacles. With the help of global guidance, a local RL planner is designed to accomplish kino-dynamic, feasible, and collision-free motion planning that incorporates dynamic obstacles within the sensing range. Specifically, to improve the network generalization, a deep V-network with an attention mechanism is designed in local RL planner and a multi-stage, multi-scenario training strategy is adopted in the training process. The contributions of this work are as follows:

- Unlike the literature [13,14,15,16,17,18,19], the method proposed in this paper can realize safe and fast path planning while satisfying the second-order kinematics model and constraints of fixed-wing UAVs;
- For the motion planning problem studied in this paper, a deep V-network based on the attention mechanism is adopted with a multi-stage, multi-scenario training strategy to improve training efficiency and network generalization. The effectiveness of the algorithm is verified by comparison simulation experiments.

## 2. Related Works

(1) Traditional motion planning algorithms for fixed-wing UAVs: Existing work on the navigation problem in cluttered environments for fixed-wing UAVs often focuses on traditional algorithms. The A* algorithm is employed to generate planning path in [13,14]. The authors in [15,16] proposed an improved potential field method to help UAVs avoid obstacles in local path planning. However, the above papers only consider the UAV as a mass point and neglect its kinematic model. The authors in [17] modeled a fixed-wing UAV as a first-order equation and defined a safe flight corridor based on its maneuvering characteristics and Dubins curve to achieve obstacle avoidance. A multi-objective optimization model was established in [20] and proposed an improved particle swarm optimization to solve the three-dimensional path planning problem in complex environments. The work proposed in [21] combined the ant colony algorithm, the self-organizing mapping algorithm, and the optimal Dubins trajectory to achieve the task assignment and path planning for multiple UAVs. Considering the kinematic model of fixed-wing UAVs as second-order systems, Ref. [22] proposed an improved RRT* algorithm to realize 3D path planning.
(2) Deep neural network-based learning methods in the field of robotics: In recent years, with the increase in computational power, deep neural network-based learning methods have shown great potential in dealing with high-dimensional and complex environmental states [23,24], especially in the field of robotics, such as robotic arms [25], wheeled and multi-legged robots [26,27,28], etc., which have been applied to some experimental real-world scenarios. For example, in [29], the authors proposed an imitation learning approach based on perceptual information to realize motion planning for UAVs, and Ref. [30] used deep learning to achieve effective 3D exploration of a vehicle in an indoor environment.
(3) Deep reinforcement learning (DRL)-based motion planning for fixed-wing UAVs: Unlike the supervised learning methods mentioned above, the RL method is applicable to sequential decision control problems, based on which agents can learn to explore strategies that maximize reward by interacting with the environment. Under the assumption that the UAV flies at a fixed altitude, Li et al. [18] designed the action space to satisfy the first-order fixed-wing UAV kinematic constraints, and implemented a deep Q-network to generate a safe path for a fixed-wing UAV. Based on the same model, Wu et al. [19] proposed a multicritical delay depth deterministic policy gradient method. Moreover, Li et al. [31] considered the second-order kinematic constraints, and designed a deep deterministic policy gradient algorithm to enable UAVs’ complete path planning in a 3D environment with only static obstacles; Yan et al. [32] established a posture assessment model to evaluate the collision risk between followers for the case with only neighboring aircraft, and designed a DRL algorithm to implement a collision-free strategy for fixed-wing UAVs.

## 3. Preliminaries

### 3.1. Problem Formulation

Consider a scenario where a fixed-wing UAV must navigate from an initial position $p_{0}$ to a goal position $p_{g}$ on the plane $R^{3}$, surrounded by a number of non-communicating UAVs and static obstacles. The control state of a fixed-wing UAV at time *t* can be represented by $\varsigma_{t}$ (defined in Section 3.2), $u_{t}$ is the control input (defined in Section 4.3.1). The next state $\varsigma_{t+1}$ can be obtained through $f\left(\varsigma_{t},u_{t}\right)$, where *f* is the kinematic model of the fixed-wing UAV (defined in Section 3.2). $O_{t}$ denotes the area occupied by the fixed-wing UAV, $O^{s}$ by static obstacles and $O_{t}^{j}$ by each surrounding UAV. We aim to drive the ego-UAV with the kinematic model *f*, to the goal position $p_{n}$ under the collision avoidance constraints and boundary constraints of control state and control input, as shown in (1a), (1c) and (1d).

We then illustrate this problem in a RL framework. At each time-step *t*, the ego-UAV first obtains its state $s_{t}$ (defined in Section 4.3.1), the set of the neighboring UAVs’ states $S_{t}=\cup_{i\in \{1,\ldots ,N\}}s_{t}^{i}$ within the local FOV and a local segment of global guidance $G^{*}$, expressed as $G_{t}^{*}$, then takes action $a_{t}$, leading to the immediate reward $R(s_{t},a_{t})$ (defined in Section 3.2) and next state $s_{t+1}=h\left(s_{t},a_{t}\right)$, under the transition model *h*. We use the superscript j∈1,…,N to denote the j-th nearby UAV within the local FOV and omit the superscript when referring to the UAV.

With the above definition, the objective is transmitted to learn a policy π for the UAV that minimizes time to goal while avoiding conflicts with neighbors and static obstacles, defined as

(1) $$\begin{array}{cc} \; & \pi^{*}=\underset{\pi }{\arg\max}E\left[\sum_{t=0}^{T}\gamma^{t\cdot v_{max}}R\left(s_{t},\pi \left(s_{t},S_{t}\right)\right)\right] \end{array}$$

(1a) $$\begin{array}{cc} \; & s.t.\;\varsigma_{t+1}=f\left(\varsigma_{t},u_{t}\right) \end{array}\;$$

(1b) $$\begin{array}{cc} \; & \;s_{T}=goal \end{array}\;$$

(1c) $$\begin{array}{cc} \; & \;O_{t}\left(\varsigma_{t}\right)\cap O_{t}^{j}=\emptyset ;O_{t}\left(\varsigma_{t}\right)\cap O^{s}=\emptyset \\ \; & \;u_{t}\in U,s_{t}\in S,\varsigma_{t}\in \Theta \end{array}\;$$

(1d) $$\begin{array}{cc} \; & \;\forall t\in \left[0,T\right],\forall j\in \left\{1,\ldots ,n\right\} \end{array}\;$$

where (1a) is the transition dynamic constraints considering the kinematic model of the fixed-wing UAV; (1b) is the terminal constraints. Note that the goal in (1b) is not exactly the goal position $p_{n}$, since $s_{T}$ is defined as the terminal state of $s_{t}$, which will be given in Section 4.3.1. (1c) is the collision avoidance constraints and *S*, *U*, and Θ are the set of admissible states, inputs, and the set of admissible control states, respectively. It is assumed that the fixed-wing UAV can obtain its current position and velocity (e.g., with on-board sensing) and the information of all the static obstacles, so it can calculate the global guidance at the start of each run. However, only the relative position and velocity of other UAVs can be observed when they are within their local FOV. Moreover, we assumed that other UAVs follow the policy that is generated by the optimal reciprocal collision avoidance (ORCA) algorithm [33].

### 3.2. Dynamics of Fixed-Wing UAV

At time-step *t*, the control state of a fixed-wing UAV can be represented by the tuple $\varsigma_{t}=\left(x_{t},y_{t},z_{t},v_{t},\theta_{t},v_{zt}\right)$, where $\left(x_{t},y_{t},z_{t}\right)$ denotes the position in 3D space; $v_{t}$ and $v_{zt}$ are the horizontal and vertical velocities relative to a inertial frame; $\theta_{t}$ denotes the heading angle.

To simplify the planning process, we decouple the horizontal and vertical motion directions of the fixed-wing UAV; then, the kinematic model of the fixed-wing UAV can be expressed as (see (1a))

(2) $$\frac{d}{dt}\left\{\begin{array}{c} x_{t}\\ y_{t}\\ z_{t}\\ v_{t}\\ \theta_{t} \end{array}\right\}=\left\{\begin{array}{c} v_{t}\cos\theta_{t}\\ v_{t}\sin\theta_{t}\\ v_{zt}\\ f_{v}\left(v_{t},u_{t}\right)\\ f_{\theta }\left(\theta_{t},\omega_{t}\right) \end{array}\right\}$$

(3) $$\begin{array}{c} f_{v}\left(v_{t},u_{t}\right)=clip\left(v_{t}+u_{t}\cdot dt,v_{\min},v_{\max}\right)\\ f_{\theta }\left(\theta_{t},\omega_{t}\right)=\theta_{t}+\omega_{t}\cdot dt\\ u_{t}\in \left[u_{\min},u_{\max}\right],\omega_{t}\in \left[\omega_{\min},\omega_{\max}\right],v_{zt}\in \left[v_{z\min},v_{z\max}\right] \end{array}$$

where $u_{t}$ and $\omega_{t}$ are the control commands.

For fixed-wing UAVs, a certain relative velocity with respect to air is necessary to generate enough lift to balance gravity. Moreover, the horizontal minimum turning radius is another limitation in fixed-wing UAV flight, resulting in constraints of heading angular velocity. To that end, three vehicle kinematic constraints are considered in this planning stage: horizontal and vertical velocity, horizontal acceleration, and heading angular velocity (which are Equation (3)).

Similarly, For each agent j∈[1,n], $p_{t}^{j}={\left[x_{t}^{j},y_{t}^{j},z_{t}^{j}\right]}^{T}\in R^{3}$ denotes its position in 3D space, $v_{t}^{j}\in R^{2}$ and $v_{zt}^{j}\in R$ its horizontal and vertical velocities at step *t* relative to a inertial frame, and $r_{j}$ the minimum radius of the ball that can completely encompass the *j*-th UAV.

## 4. Approach

In this section, we first describe the overall system structure, and then present details of our approach.

### 4.1. Systems Description

**Figure 1:**
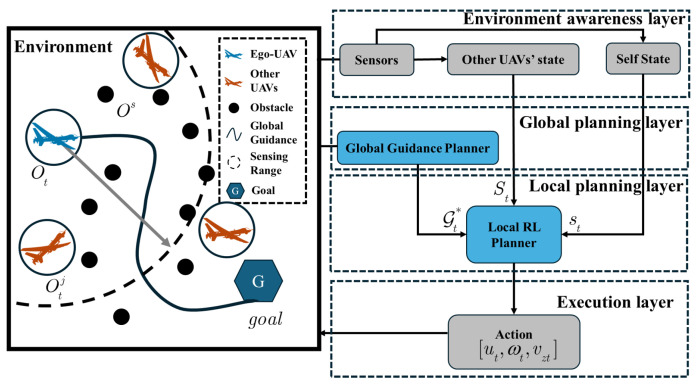
HADRL algorithm structure.

Our goal is to learn a kino-dynamic feasible and collision-free navigation policy via reinforcement learning, while relying on a local segment of global path and information within the sensing range as input, as shown in Figure 1. First, in the environment awareness layer, UAV obtains its self state and other UAVs’ information through sensors. Secondly, in the global planning layer, the global planner, which knows the information of all the static obstacles, provides a global guidance for ego-UAV. Then, in the local planning layer, a local RL planner is designed to accomplish kino-dynamic feasible and collision-free motion planning with the help of global guidance and other dynamic information within the sensing range. Finally, in the execution layer, ego-UAV executes control inputs and interacts with the environment.

It is assumed that the UAV knows the information of all the static obstacles and calculates the global guidance at the start when the scene changes. However, the trajectories of other UAVs are unknown and the ego-UAV can only obtain the current information of them when they are within its local field of view.

To that end, we adapt a hierarchical two-layer motion planning framework to tackle the navigation problem. The global planner is designed based on to a modified hybrid A* search, providing a reference guidance for ego-UAV with respect to static obstacles (Section 4.2). With the help of global guidance, a local RL planner is designed to accomplish kino-dynamic feasible and collision-free motion planning that incorporates dynamic obstacles within the sensing range (Section 4.3).

### 4.2. Global Guidance Planner

The main role of global guidance is to provide long-term global information and a safety reference path corresponding to static obstacles. With the help of global guidance, the proposed local RL planner is able to avoid potential collisions and unnecessary detours only by exploiting sensing information within a local area (e.g., a field of view).

Here, we employ a modified hybrid A* search to generate a safe and kino-dynamics feasible global path. Instead of straight-line segments, motion primitives with respect to the fixed-wing UAV dynamics described in Equations (2) and (3) are used as edges connecting two nodes. In contrast to standard hybrid A* search, the modifications are in three aspects.

#### 4.2.1. Primitive Generation

In contrast to [13,14,15,16,17,18,19], a fixed-wing UAV dynamics respecting to second-order system Equation (2) and kinematic constraints Equation (3) is considered. To achieve this, the current heading angle and horizontal velocity are added to the node information, that is, the current node is defined as $P_{k}=\left(x_{k},y_{k},\theta_{k},v_{k},P_{k-1}\right)$, where $P_{k-1}$ is the parent node. To expand the neighboring node, 11 equally spaced points within $\left[u_{min},u_{max}\right]$ and 7 equally spaced points within $\left[\omega_{min},\omega_{max}\right]$ are selected to obtain $n_{i}$ different combinations of action values. Then, the neighboring node $P_{k+1}^{i}=\left(x_{k+1}^{i},y_{k+1}^{i},\theta_{k+1}^{i},v_{k+1}^{i},P_{k}\right)$ of $P_{k}$ is calculated as

(4) $$\begin{array}{c} x_{k+1}^{i}=x_{k}+v_{k+1}^{i}\cos\theta_{k+1}^{i}\Delta t\\ y_{k+1}^{i}=y_{k}+v_{k+1}^{i}\sin\theta_{k+1}^{i}\Delta t\\ v_{k+1}^{i}=clip\left(v_{k}+u_{k+1}^{i}\Delta t\right)\\ \theta_{k+1}^{i}=\theta_{k}+\omega_{k+1}^{i}\Delta t \end{array}$$

where $i=1,\ldots ,n_{i}$,$n_{i}$ is the maximun number of neighboring nodes.

#### 4.2.2. Analytic Expansion

Inspired by reference [34], an analytic expansion scheme is induced to deal with the difficulty of having a primitive end exactly at the goal and to speed up the searching. To achieve this, Dubins curves [35,36], which are widely used in path planning for vehicles subject to turn radius constraints such as fixed-wing UAVs and underwater robots, are incorporated into the hybrid A* algorithm. Specifically, for the current node $P_{k}$, the trajectory from $P_{k}$ to $P_{g}$, which is kino-dynamics-feasible and minimal with respect to the distance is computed under Dubins curves assumption. If it satisfies collision-free condition, the searching is terminated in advance. This strategy can improve efficiency of generating global guidance path greatly, since it reduces the complexity of the search space and terminates the searching earlier.

#### 4.2.3. Cost Function

Similar to the classical A* algorithm, the notation *g* is used to represent the actual cost from the initial node $P_{0}$ to the current node $P_{k}$. As we aim to find a path that has the shortest distance and is smooth enough, *g* is calculated as

(5) $$\begin{array}{c} g=g\left(P_{k}\right)+g_{k+1,u,\omega }^{i},k=0,1,\ldots \\ g_{k+1,u,\omega }^{i}=v_{k+1}^{i}\cdot \Delta t+\alpha_{u}\left|u_{k+1}-u_{k}\right|+\alpha_{\omega }\left|\omega_{k+1}-\omega_{k}\right| \end{array}$$

In addition, the heuristic cost from Pk to Pg, *h* is calculated based on the length of the Dubins curve $l_{dk}^{g}$ as designed in the previous section, i.e., $h_{k,u,\omega }^{i}=l_{dk}^{g}$.

In summary, the flow of the hybrid A* algorithm is shown in Algorithm 1, where P and C refer to the open and closed list.

### 4.3. Local RL Planner

In this part, we aim to develop a decision-making algorithm to provide an estimate of the cost to go in dynamic environments with moving UAVs, which can inform the action, leading to higher rewards.

#### 4.3.1. RL Formulation

For an ego-UAV, the observation vector is composed of three parts: states of itself, the surrounding UAVs’ states, and a local segment of global guidance, defined as

(6) $$o_{t}=\left[s_{t},S_{t},G_{t}^{*}\right]$$

where $s_{t}$ is the ego-UAV state, $S_{t}$ is the aggregation of neighbouring UAVs’ states, N is the total number of surrounding UAVs within sensing range, $G_{t}^{*}$ is the global guidance given by Section 3.2. Specifically, each UAV’s state is parametrized as

(7) $$\begin{array}{c} s_{t}=\left[d_{gt},h_{t},v_{t},\theta_{t},v_{zt},r\right]\;\left(\mathrm{ego}-\mathrm{UAV}\right)\\ s_{t}^{j}=\left[{\bar{p}}_{jt},{\bar{v}}_{jt},r_{j},{\bar{d}}_{jt},r_{j}+r\right]\;\left(\mathrm{Other}\;\mathrm{UAVs},\;\mathrm{labelled}\;\mathrm{by}j,j=1,\ldots ,N\right) \end{array}$$

where $d_{gt}$ and $h_{t}$ denote the horizontal distance and vertical distance to the goal, respectively; ${\bar{p}}_{jt}$, ${\bar{v}}_{jt}$, and ${\bar{d}}_{jt}$ represent the relative position, velocity and distance of the jth UAV with respect to ego-UAV; *r* denote the minimum radius (size) of the ball that can encompass ego-UAV, respectively.

Here, we seek to learn the optimal policy for the ego-agent $\pi :\left(o_{t}\right)\to a_{t}$ mapping the ego-agent’s observation of the environment to a probability distribution of actions. According to (1), we consider a continuous action space $a_{t}\in R^{3}$ and define an action vector as

(8) $$a_{t}=\left[u_{t},\omega_{t},v_{zt}\right]$$

where $u_{t}\in \left[u_{\min},u_{\max}\right]$, $\omega_{t}\in \left[\omega_{\min},\omega_{\max}\right]$, $v_{zt}\in \left[v_{z\min},v_{z\max}\right]$.

For simplicity, it is assumed that the UAV is at maximum vertical speed when it is far from the goal position. In other words, $v_{zt}$ is updated as below:

(9) $$v_{zt}=\left\{\begin{array}{c} v_{z\max},h_{t}>v_{z\max}\cdot \Delta t\\ \frac{h_{t}}{\Delta t},v_{z\min}\cdot \Delta t\leq h_{t}\leq v_{z\max}\cdot \Delta t\\ v_{z\min},h_{t}<v_{z\min}\cdot \Delta t \end{array}\right.$$

For this task, a compound reward function is designed to feed back signals considering multiple subobjectives and generate dense rewards to promote convergence. More specifically, at each step, the reward function is given by

(10) $$R_{t}=R_{1t}+R_{2t}+R_{3t}$$

where $R_{1t}$, $R_{2t}$, and $R_{3t}$ are designed for the purpose of collision avoidance, goal arrival, and global information awareness, respectively.

Firstly, the collision avoidance reward $R_{1t}$ is obtained by

(11) $$R_{1t}=\left\{\begin{array}{c} r_{collision},{\bar{d}}_{t}\leq 0\\ r_{collision}+\alpha_{1}\cdot {\bar{d}}_{t},0<{\bar{d}}_{t}\leq d_{safe}\\ 0,{\bar{d}}_{t}>d_{safe} \end{array}\right.$$

where $r_{collision}$ is a large negative penalty for collision, ${\bar{d}}_{t}$ is the minimum sensing distance to the nearest obstacle in real time, and $\alpha_{1}$ is a small positive number. The second line makes the UAV alert to the nearest obstacle, assigned a penalty value if ${\bar{d}}_{t}$ is less than a predefined threshold $d_{safe}$.

Secondly, the goal arrival reward $R_{2t}$ is computed by

(12) $$R_{2t}=\left\{\begin{array}{c} r_{success},d_{gt}\leq d_{\min}\\ \alpha_{2}\left(d_{gt-1}-d_{gt}\right),d_{gt}>d_{\min}\\ r_{fail},t\geq t_{\max} \end{array}\right.$$

where $r_{success}$ is a large positive reward for arrival, while $\alpha_{2}$ is a small positive number used to motivate the UAV to move towards the goal when navigating, and $d_{min}$ is the Euclidean distance from the UAV to its goal UAV when it is identified as reaching the goal, since the fixed-wing UAV may not be able to reach the goal accurately.

Thirdly, to encourage the UAV to take the global information into account, while simultaneously not following the global guidance strictly, $R_{3}\left(t\right)$ is computed by

(13) $$R_{3t}=\left\{\begin{array}{c} 0,d_{at}\leq d_{a\max}\\ -\alpha_{3}\cdot \left(d_{a\max}-d_{at}\right),d_{at}>d_{a\max} \end{array}\right.$$

where $d_{at}$ denotes the distance between the current UAV and the global guidance path at the time of *t*; $\alpha_{3}$ is a small positive number; $d_{amax}$ is the predefined allowable deviation distance.

#### 4.3.2. Policy Network Architecture

**Figure 2:**
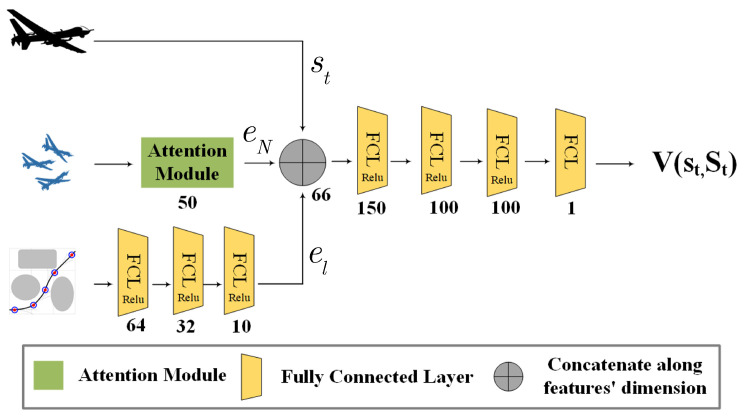
HADRL algorithm network structure.

Figure 2 depicts the network structure of HADRL. Here is the description in detail. Considering that $o_{t}$ consists of three groups, i.e., ($s_{t}$, $S_{t}$, $G_{t}^{*}$), we use two embedding functions to encode them separately. Through three fully connected (FC) layers, the embeddings of local segment of global guidance $G_{t}^{*}$ can be easily represented as

(14) $$e_{l}=FC_{l}\left(G_{t}^{*}\right)$$

**Figure 3:**
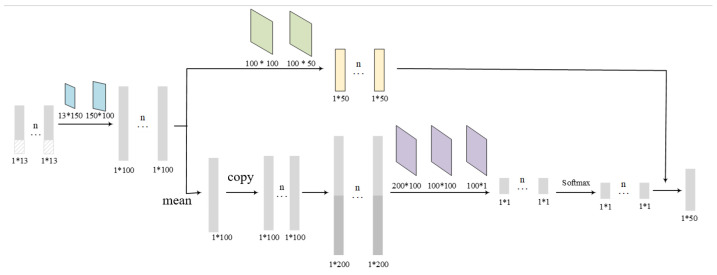
Attention mechanism module. Different color is used to illustrate different placement of linear layers for clarify.

As the numbers of surrounding UAVs in field of view are uncertain, we customize an attention embedding block to aggregate the states of the neighbors $S_{t}$. As shown in Figure 3, the aggregated embedding of $e_{N}$ is computed by

(15) $$e_{N}=Attention\left(S_{t}\right)=\sum_{j\in N}\xi_{N}^{j}FC_{N1}\left(s_{t}^{j}\right)$$

The attention weight $\xi_{N}^{j}$ is calculated as

(16) $$\begin{array}{c} \xi_{N}^{j}=\frac{\exp(q_{N}^{j})}{\sum_{j}\exp(q_{N}^{j})}\\ q_{N}^{j}=FC_{N2}(\frac{\sum_{j}FC_{N3}\left(s_{j}\right)}{N}\|s_{j}) \end{array}$$

As shown in Figure 2, we concatenate the fixed-length representation of the other agent’s states with the ego-agent’s state and the embedding of global guidance to create a joint state representation. This representation vector is fed to four fully connected layers (FCL). The output estimates the state-value function $V^{\pi }\left(S_{t}\right)=E_{S_{t+1:\infty }}\left[\sum_{l=0}^{\infty }r_{t+l}\right]$, with a linear activation function.

### 4.4. Hybrid A* Deep V Network (HADVN) Algorithm

An agent is unlikely to reach a goal position with an initial RL policy. Hence, during the pretraining phase, we use the ORCA as an expert and perform supervised training to train the policy and value function parameters for $n_{ORCA}$ steps. By setting the current state and using the ORCA, we obtain a sequence of state, action, next state, and reward in a buffer $B\leftarrow \{o_{k},a_{k},o_{k+1},r_{k}\}$. Then, we compute advantage estimates and perform a supervised training step:

(17) $$\theta_{k+1}^{V}=\arg\min_{\theta^{V}}E_{\left(a_{k},o_{k},r_{k}\right)∼D_{ORCA}}\left[\left\|V_{\theta }\left(o_{k}\right)-V_{k}^{trag}\right\|\right]$$

where $\theta^{V}$ is the value function and policy parameters.

In this work, we train the policy using the deep V-learning algorithm proposed by Chen C et al. [37]. The reasons for choosing a deep V-network instead of a Q-network in this paper are as follows: first, compared with deep Q-networks, the structure of V-networks are simpler, since their output is no longer different combinations of the action space, but can be simplified to a single node; second, in the supervised learning, the optimal policy can be computed faster, and the data-processing operations can be simplified; third, since the upper and lower bounds of the action quantities (acceleration and angular velocity) are different in this paper, the optimal policy can be computed quickly. We propose to jointly train the guidance policy $V^{\pi }$ with the ORCA algorithm. Algorithm 1 describes the proposed training strategy and has two main phases: supervised and RL training. First, we randomly initialize the value function parameters $\theta^{V}$. Then, a multi-scene multi-stage training framework is used for the training of the network. More details about the different training scenarios considered are given in Section 5.
**Algorithm 1** Hybrid A* deep V-network (HADVN) algorithm**Input:** target value network, number of supervised and RL training episodes $\left\{n_{ORCA},n_{episodes}\right\}$, size of mini-batch $n_{mini-batch}$
**Output:** Optimal value network $V^{\pi }$ 1:Initialize the optimal value network *V*, the target value network $\hat{V}$ 2:Initialize the open list P and close list C, add $P_{0}$ to P 3:**while** P not empty **do** 4:    From P take the node with the smallest total cost *f* and record it as the current node $P_{k}$, add $P_{k}$ to C; 5:    **if** $\left\|\left(x_{k},y_{k}\right)-\left(x_{g},y_{g}\right)\right\|\leq d_{\min}$ **then** 6:        **return** $\left\{P_{0},\cdots ,P_{k}\right\}$; 7:    **else** 8:        generate Dubins curves $l_{dk}^{g}$ from $P_{k}$ to $P_{g}$; 9:    **end if**10:    **if** $l_{dk}^{g}$ satisfies the no-collision condition, **then**11:        generate path points $\left\{P_{k+1},\ldots P_{g}\right\}$ on $l_{dk}^{g}$, **return** $\left\{P_{0},\cdots ,P_{g}\right\}$;12:    **end if**13:    Based on discretized action combinations $\left(u_{k+1}^{i},\omega_{k+1}^{i}\right)$ and Equation (4), generate the set of neighboring nodes $\left\{P_{k+1}^{i}\right\}$ of $P_{k}$, where $i=1,\cdots ,n_{i}$;14:    **for** $P_{k+1}^{i}$**in** $\left\{P_{k+1}^{i}\right\}$ **do**15:        **if** $P_{k+1}^{i}$ is beyond the map boundary, close to an obstacle or already in C in, **then**16:           **continue**;17:        **end if**18:        Using Equation (5), calculate $g=g\left(P_{k}\right)+g_{k+1,u,\omega }^{i}$;19:        **if** $P_{k+1}^{i}$**not in** O, **then**20:           add $P_{k+1}^{i}$ to O;21:        **else if** $g\geq g\left(P_{k+1}^{i}\right)$, **then**22:           **continue**;23:        **end if**24:    **end for**25:    $g\left(P_{k+1}^{i}\right)\leftarrow g$, change the parent node of $P_{k+1}^{i}$ to $P_{k}$;26:    Calculate the total cost *f* of the node $P_{k+1}^{i}$;27:**end while**28:Generate $G^{*}$ from $\left\{P_{0},\cdots ,P_{k}\right\}$;29:Back to step 2 if training scenario is changed;30:**while** $episode<n_{episodes}$ **do**31:    Initialize B and states;32:    **for** $k=0,\ldots ,n_{mini-batch}$ **do**33:        **if** $episode\leq n_{ORCA}$ **then**34:           using ORCA to get $a_{k}$;35:        **else**36:           $a_{k}=\arg\max_{a_{k}\in A}R\left(o_{k},a_{k}\right)+\gamma^{\Delta t\cdot v_{\max}}V\left(o_{k+1}\right)$;37:        **end if**38:        $\left\{o_{k},a_{k},o_{k+1},r_{k},done\right\}=step\left(o_{k},a_{k}\right)$;39:        Store $B\leftarrow \{o_{k},a_{k},o_{k+1},r_{k}\}$;40:        **if** done **then**41:           episode+=1, updating the target network $\hat{V}\leftarrow V$;42:        **end if**43:    **end for**44:    **if** $episode<n_{episodes}$ **then**45:        Supervised training46:    **else**47:        Randomly take a $\left\{o_{k},a_{k},o_{k+1},r_{k}\right\}$ from B;48:        Calculate of the target value $y_{k}=r_{k}+\gamma^{\Delta t\cdot v_{\max}}\hat{V}\left(o_{k+1}\right)$;49:        Update the optimal value network *V* by gradient descent;50:    **end if**51:**end while**


## 5. Results

This section quantifies the performance throughout the training procedure, and compares the proposed method against the baseline approach, socially attentive reinforcement learning (SARL) algorithm [37], which adopts LSTM to deal with an uncertain number of dynamic obstacles in the environment. Although SARL only considers the first-order kinematic model of the mobile robot, in the comparative experiments, we improve both its pretraining and subsequent incremental training sessions to make it applicable to the generation of motion trajectories of fixed-wing UAVs.

### 5.1. Experimental Setup

The proposed training algorithm builds upon deep V-learning. We used a laptop with a 12th Intel Core i9 and 32 GB of RAM for training. Hyperparameter values are summarized in Table 1.

### 5.2. Training Procedure

To train and evaluate our method, we selected a multi-scene, multi-stage training framework used for the training of the network, which can effectively increase the convergence speed of the network and further improve the generalization ability and effectiveness of the strategy, compared to training only in a single complex environment [38,39,40]. Here, we consider three different scenarios. In the first scenario, there are no static obstacles, and the number of static obstacles is increased in the second and third scenarios. The obstacles are defined as cylinders of equal height and radius with randomly generated locations. To increase the difficulty of obstacle and collision avoidance, the UAV to be planned is randomly distributed with other adjacent UAVs on a horizontal circle, and the goal position of the UAV to be planned is randomly placed on the other side of the circle.

Prior to reinforcement learning training, the network was first pretrained using the ORCA algorithm. In the pretraining phase, all UAV strategies were given by ORCA, 3000 rounds of experience were collected in the experience pool, and 50 rounds of training were performed on the optimal strategy network with a learning rate of 0.001.

**Figure 4:**
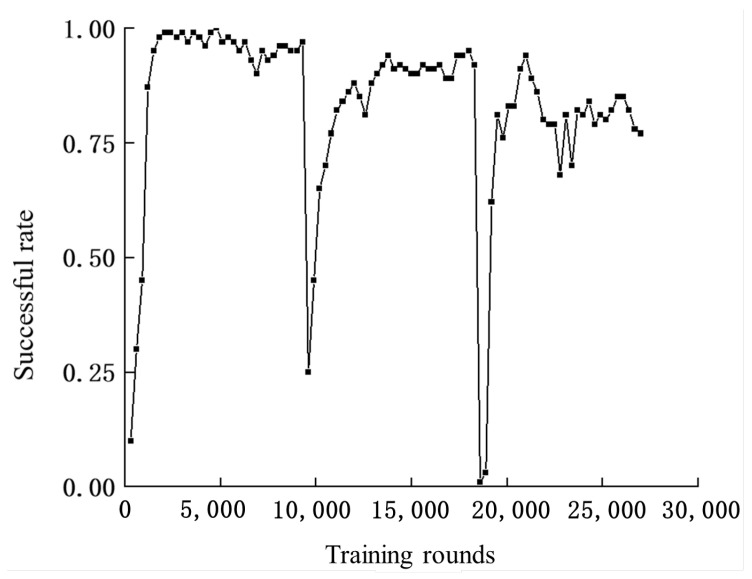
Number of training rounds—average reward curve.

Training is completed in a total of three phases, corresponding to three different scenarios, with 9000 training rounds for each scenario. Every 300 rounds, the experience pool is emptied and the success rate of the algorithm in the current environment is tested. The experiments show that staged training can significantly improve the speed of convergence, as shown in Figure 4.

### 5.3. Qualitative Analysis

**Figure 5:**
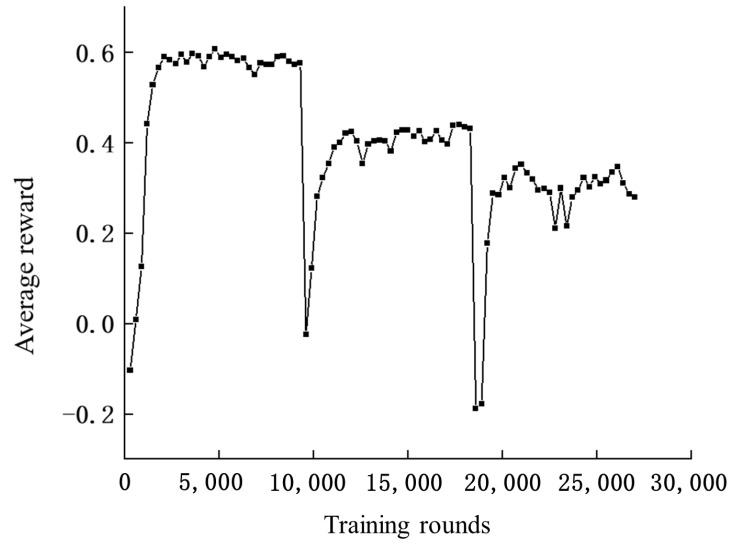
Number of training rounds—success rate curve.

This section analyzes the trained method. As shown in Figure 4 and Figure 5, the average reward is about 0.6 and the success rate is close to 0.98 at 2100 training rounds, indicating that the algorithm has learned the ability to avoid obstacles in the absence of static obstacles. When the environment is changed in 9000 rounds, the algorithm’s success rate and average reward show a significant drop, indicating that the ability to avoid static obstacles has not been learned. After training to the 13,800th round, the average reward for the second scenario is about 0.4 and the success rate is around 0.9. In the third scenario, after training to round 21,000, the average reward for the second scenario basically is about 0.3, with a success rate of around 0.8.

As shown in Figure 5, the average reward becomes lower as the static obstacles in the environment increase. This is because UAVs have to take a longer detour to avoid obstacles and collisions.

**Figure 6:**
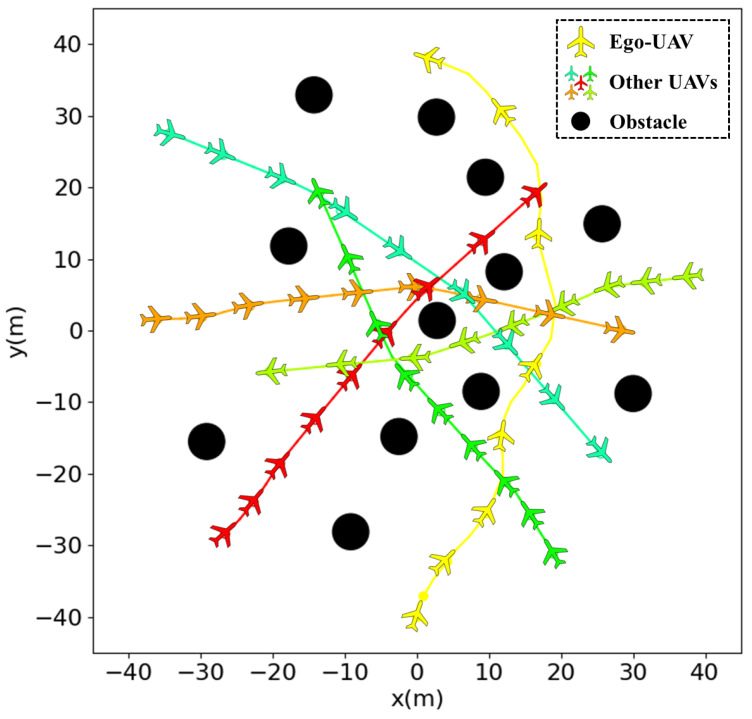
Results of trained module. In yellow is depicted the trajectory of robot; in other colors are the other UAVs; in black are the obstacles.

**Figure 7:**
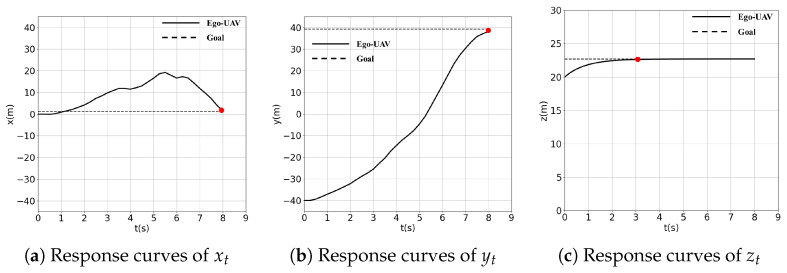
The $x_{t}$, $y_{t}$, $z_{t}$ responses. The dotted line represents the goal position.

Figure 6 gives one planning episode from top view: in yellow is depicted the trajectory of the robot; in other colors are the other UAVs; in black are the obstacles; and the time interval between each UAV is the same. Figure 7 plots the x, y, z responses of the ego-UAV. The solid line represents the curves of the ego-UAV, while the dashed line gives the goal position, from which we can see that the UAV successfully avoids all static obstacles and other UAVs while reaching the goal safely, as marked by the red dot. When using our approach, the robot initiates a collision avoidance maneuver early enough to lead to a smooth trajectory and faster arrival at the goal.

**Figure 8:**
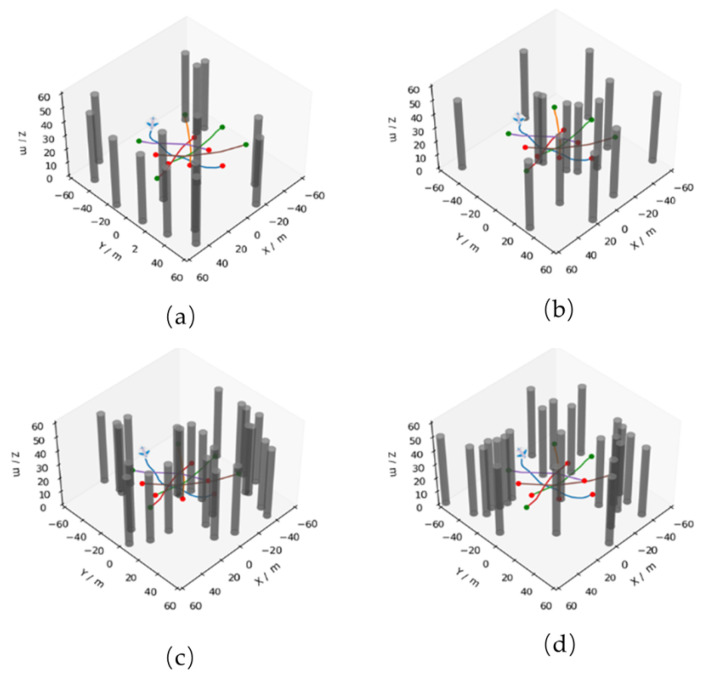
Planning result under random obstacle environment. (**a**,**b**) are the test results with the randomly generated scene with 15 obstacles, while (**c**,**d**) are the test results with the randomly generated scene with 25 obstacles, the green dot represents the starting position and the red dot represents the target position.

The paths planned in a random obstacle environment are tested at the end of training using the HADVN algorithm, as shown in Figure 8. It can be observed that the network has learned the experience of avoiding randomly generated static obstacles and other UAVs.

To simulate the complicated unknown disturbances, random Gaussian noises that affected the fixed-wing UAV dynamics are considered. We set random Gaussian disturbance as in [41], and a mean of 0 as well as a covariance of 0.5, for the x or y position, respectively. We run the trained HADRL in three different scenarios with the obstacle number as 10, 15, and 20, respectively, and record the results of 100 episodes, given in Table 2. The success rate is used to evaluate the performance, which is the proportion that ego-UAV reaches its goal without a collision. We can conclude that our approach demonstrates a good performance in the presence of random Gaussian disturbances without extra training.

### 5.4. Performance Results

**Figure 9:**
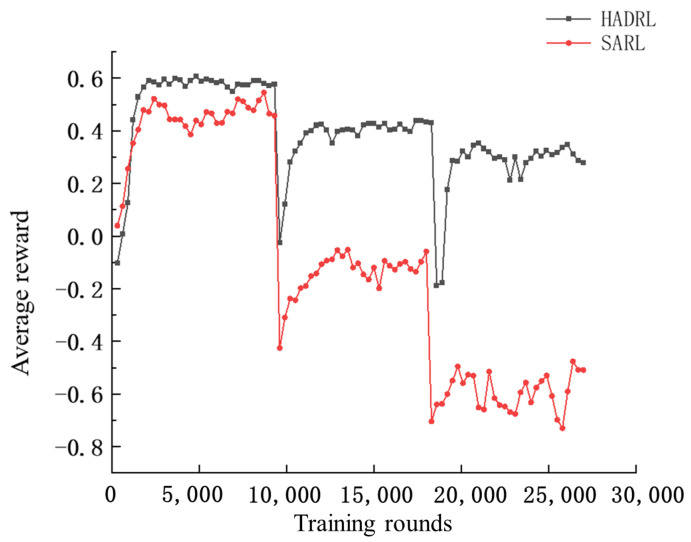
Comparison of the number of training rounds and average reward curves.

**Figure 10:**
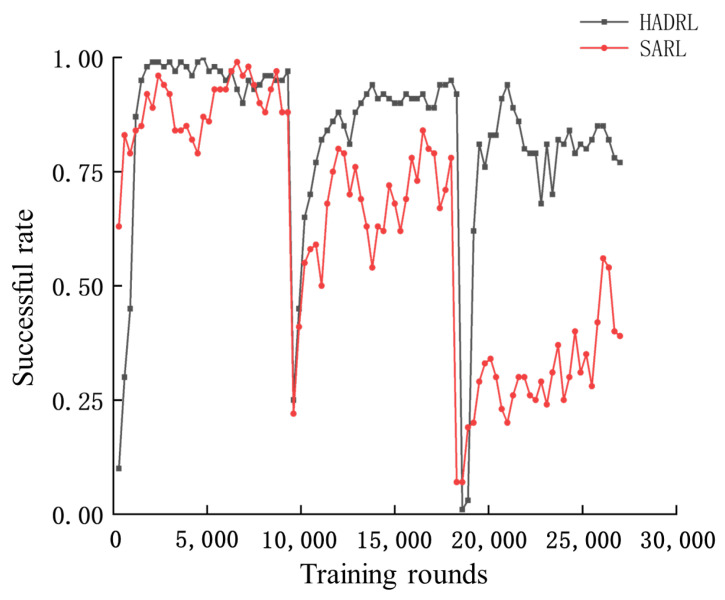
Comparison of training rounds–success rate curves for the two algorithms.

This section compares it with the baseline method, SARL. The simulation results are shown in Figure 9 and Figure 10, it can be seen that during the training process of the network, in the first stage, the difference between the average reward and success rate of the SARL algorithm and the HADRL algorithm is not very large, and the SARL algorithm already has a high success rate after pretraining the initial network, indicating that the SARL algorithm can plan a collision-free and safe trajectory in the absence of static obstacles. However, in the second and third phases, the average reward and success rate of the SARL algorithm decrease significantly, while our proposed HADRL algorithm is able to learn and adapt to more complex environments very quickly with the help of global guidance.

Moreover, to illustrate the advantages of our proposed HADRL algorithm, comparative simulations are performed, with the ORCA and SARL algorithms. The scenarios—which are the same as in Section 5.3—and the results of 100 episodes are recorded. Two metrics—success rate and execution time—are used to evaluate the performance, where execution time is the time algorithm used to reach the goal. The results are shown in Table 3 and Table 4. It is observed that HADRL has a higher success rate in three scenarios. As for the execution time, HADRL and SARL show the same performance, which is better than ORCA. Hence, we can conclude that our algorithm performs better in terms of computational efficiency and scalability.

## 6. Conclusions

For complex environments with static obstacles and other flying UAVs, this paper fully considers the second-order kinematic model and constraints, and proposes a HADVN motion planning algorithm based on global path guidance and a local RL planner so that fixed-wing UAVs can reach the target point safely within a preset time. Unlike the widely used deep Q-networks, our approach employs a deep V-network to deal with the continuous motion space. In addition, considering the variable number of neighboring UAVs in the sensing range of the UAV, an attention mechanism is added to the front end of the network to improve the network generalization. Finally, a multi-stage, multi-scene training strategy is used in training process and the effectiveness of the algorithm is verified by simulation experiments.

## Figures and Tables

**Table 1:** Hyperparameters.

Parameters	Value
Discount factor	0.9
Learning rate	0.001
Number of training rounds	300
$n_{ORCA}$	5000
$n_{episodes}$	30,000
$n_{mini-batch}$	100
$r_{collision}$	−0.25
$r_{success}$	1
$r_{fail}$	−0.1
$\alpha_{1}$	0.05
$\alpha_{2}$	0.1
$\alpha_{3}$	0.01
$\alpha_{u}$	0.01
$\alpha_{\omega }$	0.01
$d_{safe}$	5 m
$d_{\min}$	1 m
$d_{a\max}$	10 m
Δt	0.25 s
$t_{\max}$	25 s

**Table 2:** Comparison of success rate with random Gaussian noises.

Methods	HADRL (without Noises)	HADRL (with Noises in x Position)	HADRL (with Noises in y Position)
Scenario 1	0.86	0.72	0.71
Scenario 2	0.79	0.65	0.63
Scenario 3	0.76	0.60	0.61

**Table 3:** Comparison of success rate with other planning strategies.

Methods	ORCA	SARL	HADRL
Scenario 1	0.63	0.78	0.86
Scenario 2	0.55	0.62	0.79
Scenario 3	0.43	0.57	0.76

**Table 4:** Comparison of execution time with other planning strategies.

Methods	ORCA	SARL	HADRL
Scenario 1	0.32	0.12	0.13
Scenario 2	0.35	0.13	0.14
Scenario 3	0.36	0.13	0.16

## Data Availability

Data are contained within the article.

## Abbreviations

The following abbreviations are used in this manuscript:

UAV	Unmanned Aerial Vehicle
HADVN	Hybrid A* Deep V-Network
RRT	Rapidly-exploring Random Tree
FOV	Field of View
ORCA	Optimal Reciprocal Collision Avoidance
SARL	Socially Attentive Reinforcement Learning
